# Association of the peripheral blood levels of circulating microRNAs with both recurrent miscarriage and the outcomes of embryo transfer in an in vitro fertilization process

**DOI:** 10.1186/s12967-018-1556-x

**Published:** 2018-07-04

**Authors:** Qian Yang, Wen-Wen Gu, Yan Gu, Na-Na Yan, Yan-Yan Mao, Xing-Xing Zhen, Jian-Mei Wang, Jing Yang, Hui-Juan Shi, Xuan Zhang, Jian Wang

**Affiliations:** 1 Key Laboratory of Reproduction Regulation of NHFPC, Shanghai Institute of Planned Parenthood Research, Pharmacy School, Fudan University, Shanghai, China; 2 The Second Hospital of Tianjin Medical University, Tianjin, China; 3 Renmin Hospital of Wuhan University, Wuhan, China

**Keywords:** Circulating microRNA, Peripheral blood, Recurrent miscarriage, Embryo transfer, In vitro fertilization

## Abstract

**Background:**

Implantation failure is not only a major cause of early pregnancy loss, but it is also an obstacle to assisted reproductive technologies. The identification of potential circulating biomarkers for recurrent miscarriage (RM) and/or recurrent implantation failure would contribute to the development of novel diagnosis and prediction techniques.

**Methods:**

MiR (miR-23a-3p, 27a-3p, 29a-3p, 100-5p, 127-3p and 486-5p) expression in the villi, decidual tissues and peripheral blood plasma and serum were validated by qPCR, and the localization of miRs in the villi and decidual tissues of RM and normal pregnancy (NP) women were detected by in situ hybridization. The invasiveness of HTR8/SVneo cells was determined using a Transwell assay. The predictive values of miRs for RM and the outcome of IVF-ET were respectively calculated by the receiver operating characteristic analysis.

**Results:**

The signals of six miRs were observed in the villi and decidual tissues of RM and NP women. The villus miR-27a-3p, miR-29a-3p and miR-100-5p were significantly up-regulated, whereas miR-127-3p and miR-486-5p appeared to be down-regulated in RM women compared to NP women. The invasiveness of HTR8/SVneo cells transfected with miR-23a-3p mimics was evidently weakened, whereas that of cells transfected with miR-127-3p mimics was obviously enhanced. The peripheral blood plasma levels of miR-27a-3p, miR-29a-3p, miR-100-5p and miR-127-3p were significantly increased, whereas that of miR-486-5p was remarkably decreased in RM compared to NP women. By contrast, serum miR-23a-3p and miR-127-3p were significantly decreased, whereas that of miR-486-5p was remarkably increased. The combination of six plasma miRs levels discriminated RM with a sensitivity of 100% and a specificity of 83.3%, whereas that of six serum miRs levels showed a sensitivity of 78.3% and a specificity of 93.1%. In the IVF-ET cohort, the significantly decreased peripheral blood plasma levels of miR-23a-3p, miR-27a-3p, miR-100-5p and miR-127-3p, and the serum levels of miR-100-5p and miR-486-5p, in addition to the significantly increased serum level of miR-27a-3p, were found to be associated with the failure of ET. Moreover, the combination of plasma miR-23a-3p, miR-27a-3p, miR-29a-3p, miR-100-5p, miR-127-3p and miR-486-5p levels discriminated the outcome of IVF-ET with a sensitivity of 68.1% and a specificity of 54.1%, whereas the combination of plasma miR-127-3p and miR-486-5p levels showed a sensitivity of 50.0% and a specificity of 75.3%.

**Conclusions:**

Circulating miR-23a-3p, miR-27a-3p, miR-29a-3p, miR-100-5p, miR-127-3p and miR-486-5 might be involved in RM pathogenesis and present potential diagnostic biomarkers for RM. Meanwhile, these miRs, in particular miR-127-3p and miR-486-5p, provide promising prediction indexes for the outcomes of IVF-ET.

**Electronic supplementary material:**

The online version of this article (10.1186/s12967-018-1556-x) contains supplementary material, which is available to authorized users.

## Background

Successful embryo implantation, which is a crucial step for the establishment and maintenance of pregnancy in mammals, requires a harmonized interaction between invaded blastocysts and receptive endometrium [1]. Implantation failure is not only a major cause of early pregnancy loss, but it is also an obstacle to the significant improvement of assisted reproductive technologies [2, 3].

Recurrent miscarriage (RM) can be defined as 2 or more consecutive pregnancy losses prior to the 20th week of gestation in humans. RM has an incidence of 1–2% in pregnant women, and the etiology of 68% of RM cases is unexplained [4]. Because the exact pathogenic mechanisms underlying RM remain unclear, its clinical management still lacks powerful means of diagnosis and prediction and effective therapeutic techniques [5]. The widespread application of assisted reproductive technologies, particularly in vitro fertilization (IVF), has benefited numerous infertility patients over the past decades; however, implantation failure after embryo transfer (ET), in particular recurrent implantation failure, has fettered the further advance of the IVF success rate [6, 7]. Thus, the identification and evaluation of potential circulating biomarkers for RM and/or recurrent implantation failure would undoubtedly contribute to the development of novel diagnosis and prediction techniques.

MicroRNAs (miRs) are single-stranded small non-coding RNA sequences of usually 19–25 nucleotides (nts) in length that participate in the post-transcriptional regulation of gene expression in a variety of biological and physiological processes [8]. It has been widely reported that miRs are involved in embryo implantation, and the dysfunctions of miRs are associated with implantation failure, resulting in RM and recurrent implantation failure [9, 10]. In particular, because miRs are extracellularly stable and can be detected in human plasma, and because the alteration of such circulating miRs might occur in response to physiological or pathological status [11], they are therefore identified as a new class of non-invasive biomarkers.

Recently, we established RM-related villus miR profiles by deep-sequencing identification using three pairs of tissue samples collected from RM patients and well-matched normal pregnancy (NP) women. A number of miRs were screened to potentially be differentially expressed in the villus tissues of RM patients; however, only a small part of them that met our designated criteria of P < 0.05 and fold change > 1.5 (or 2.0) were reported, including miR-100-5p and miR-486-5p [12, 13]. By searching the human serum database (http://www.exiqon.com/ plate-layout-files), we found that in addition to miR-100-5p and miR-486-5p, another four miRs, miR-23a-3p, miR-27a-3p, miR-29a-3p and miR-127-3p, which were also screened to be differentially expressed in RM patients with fold changes ranging from 1.1 to 1.5, and P < 0.05 (Additional file 1: Table S1), are presented in human peripheral blood. More interestingly, miR-23a-3p [14], miR-27a-3p [15], miR-29a-3p [16] and miR-127-3p [17] have been reported by other labs to be involved in human pathological pregnancy.

Thus, in the present study, the differential expressions of these six miRs (miR-23a-3p, miR-27a-3p, miR-29a-3p, miR-100-5p, miR-127-3p and miR-486-5p) in the villi of RM patients were validated by real-time quantitative PCR, and their expression localization in the villus and decidual tissues of RM patients and NP women were also determined by in situ hybridization. The effects of their over-expressions on the invasive activities of HTR8/SVneo were individually assessed by the transfection of their specific mimics in vitro. Finally, their plasma or serum levels in peripheral blood were detected and compared between RM patients and NP women and between pregnant and non-pregnant women undergoing IVF treatment, with the aim of identifying circulating miRs biomarkers related to both RM and the outcomes of IVF-ET.

## Methods

### Sample collection

Human villus and decidual tissues and peripheral blood samples of RM patients and NP women were collected from April to August 2017 at the Department of Gynecology and Obstetrics, the Second Hospital of Tianjin Medical University, Tianjin, China. The current pregnancy losses of RM patients were objectively confirmed by transvaginal ultrasound examination. Classical risk factors including abnormal parental karyotypes, uterine anatomical abnormalities, infectious diseases, luteal phase defects, diabetes mellitus, thyroid dysfunction, and hyperprolactinemia were excluded. In parallel, NP women, who had no history of miscarriage and were undergoing legal elective abortions, were enrolled and checked for classical risk factors for early pregnancy losses. All samples were collected after informed consent was obtained. Then, the decidual and villus tissues were frozen and stored at − 80 °C. The venous blood plasma and serum samples were freshly prepared and subsequently stored at − 80 °C.

Peripheral blood samples of IVF-ET women were collected from June to July 2017 at the Reproductive Medicine Center, Renmin Hospital of Wuhan University, Hubei Province, China. Participants who were undergoing IVF with ICSI due to male factor infertility were enrolled, and peripheral blood samples were collected prior to ET on the day of ET after informed consent was obtained. The participants were followed up till the end of the first trimester to observe the outcome of the current ET treatment.

### RNA extraction from villus tissues

Total RNA was extracted respectively from five pairs of villus samples from RM patients and NP women using TRIzol reagent according to the manufacturer’s protocol (Invitrogen, Carlsbad, CA). The concentration of total RNA products was measured by NanoDrop (Thermo Scientific, Wilmington, DE), and RNA integrity was checked with a Bioanalyzer2100 (Agilent, Santa Clara, CA).

### Real-time quantitative PCR for MiRs in placental villus tissues

Total RNA products extracted from villus tissues were used to validate the differential expression of miRs between the RM patients and NP women by real-time quantitative PCR analysis. Total RNA was reversely transcribed using miRNA specific reverse primers (Ribobio, Guangzhou, China) to obtain cDNA. Real-time PCR was performed using the FastStart Universal SYBR Green Master Kit (Roche Diagnostics, USA) according to the manufacturer’s description and was analyzed using an ABI 7900 HT (Applied Biosystems, Foster, CA). All miRs assay primers were purchased commercially (Ribobio). Primer efficiencies were determined by the standard curve. The relative expression of miRs was calculated by the efficiency-corrected ΔΔCt method and normalized to the endogenous control snRNA U6. Each sample in each group was measured in triplicate, and the experiment was repeated at least three times.

### In situ hybridization

Two pairs of villus and decidual samples (RM1 and RM2 in the RM group and NP1 and NP2 in the NP group) were chosen randomly for the in situ hybridization analysis. Frozen 10-μm serial sections were rehydrated and fixed in 4% paraformaldehyde for 20 min, treated with protease K for 5 min at room temperature, and followed by hybridization with LNA microRNA probes (Exiqon, Copenhagen, Denmark) specifically against miRNAs at 55 °C overnight. The sections were sequentially washed in 5×, 2×, and 0.2× saline-sodium citrate buffer. After blocking, the sections were incubated with alkaline phosphatase–conjugated anti-digoxin antibodies (1:200, Roche, Indianapolis, IN) at 4 °C overnight. BCIP/NBT (Promega, Madison, WI) was used as the substrate to visualize the stained signals according to the manufacturer’s instructions. The scramble miRNA (non-specific control) probe was used as the negative control.

After that, the human decidua slides were incubated with HLA-G antibodies (Abcam, 1:800) or IgG antibodies at 4 °C overnight to identify which cell type expresses these miRNAs in decidua. Further incubation with horseradish peroxidase-conjugated secondary antibodies (Vector, 1:500) was visualized with DAB (Dako Cytomation, Glostrup, Denmark) solution containing 0.03% H_2_O_2_ as the substrate. The human villi and decidua slides were counterstained with Nuclear Fast Red (Beijing Dingguo Changsheng Biotechnology, Beijing, China) and mounted with neutral balsam. The probe sequences are presented in Additional file 1: Table S2.

### HTR-8/SVneo cell culture and treatment

The HTR8/SVneo trophoblast cell line was derived from human first-trimester placenta [18]. The cells were kindly provided by Dr. Hongmei Wang, Institute of Zoology, Chinese Academy of Sciences, China, and cultured in RPMI-1640 medium supplemented with fetal bovine serum (FBS; Gibco, Carlsbad, CA), 100 U/ml penicillin (Gibco) and 100 µg/ml streptomycin (Gibco) under standard culture conditions (37 °C in a 5% humidified CO_2_ incubator). The cells used for transfection were plated 30,000 per well in the 24-well plate (Corning, NY, USA) using 500 μl of medium. The mimics and inhibitors of miRs and their negative control (mimic-NC/inhibitor-NC) were purchased from Ribobio. When the cells reached 70% confluence, they were transiently transfected by the mimics (10 µM) and mimic-NC (10 µM), respectively, using Lipofectamine™ 2000 (Thermo scientific) according to the manufacturer’s instruction. After 6 h, the cells were washed with serum-free and antibiotics culture medium and continued being cultured in complete medium (1 ml/well) overnight.

### Transwell assay of HTR-8/SVneo cell invasiveness

Cell invasion was quantified using a BD BioCoat™ Matrigel™ Invasion Chamber (Corning) according to the manufacturer’s instructions. Briefly, warm culture medium was added to a 24-well plate, and 8-μm pore Transwell inserts were plated into the wells and rehydrated for 2 h at 37 °C. After 24 h of miRNA mimic or inhibitor transfection, the HTR8/SVneo cells were harvested with serum-free medium, 2 × 10^5^ cells were seeded into the upper compartment of the prepared inserts, and medium supplemented with 25% FBS (Gibco) was added to the lower compartment to induce migration. After 24 h of incubation at 37 °C with 5% CO_2_, the cells remaining inside the inserts were removed using a cotton swab. The membranes were then fixed with 4% paraformaldehyde, stained with 0.1% crystal violet, washed with ddH_2_O and observed under the microscope. Then, the cells were lysed with methanol, and the absorbance at a wavelength of 560 nm was measured using a UV spectrophotometer for quantification. The experiments in each group were repeated three times under the same conditions.

### Blood plasma and serum separation and RNA extraction

Whole venous blood samples used for plasma separation were collected in EDTA-containing tubes and then immediately centrifuged at 1600×*g* for 10 min to separate the plasma samples. The plasma samples were carefully transferred into new tubes and stored at − 80 °C until use. To prepare the serum samples, the whole blood was collected in blood collection tubes without anticoagulants and placed at room temperature (15–25 °C) for 30 min to complete clotting. Then, the blood sample tubes were centrifuged for 10 min at 1900×*g* (3000 rpm) under 4 °C. The yellow upper serum phase was carefully transferred to a new tube without disturbing the intermediate buffy coat layer (containing white blood cells and platelets) and kept frozen in aliquots at − 80 °C. Total RNA was extracted from the plasma or serum using a miRNeasy Serum/Plasma Kit (Qiagen, Suzhou, China) following the manufacturer’s instructions. The quantity and quality of obtained miRNA was measured with a NanoDrop ND-1000 Spectrophotometer (Thermo scientific).

### Real-time quantitative PCR for MiRs in plasma and serum

Total RNAs extracted from plasma and serum were reversely transcribed using miRNA specific reverse primer (Ribobio) to obtain cDNA. Real-time PCR was performed using the FastStart Universal SYBR Green Master (Roche Diagnostics, Basel, Switzerland) according to the manufacturer’s description and analyzed using an ABI 7900 HT (Applied Biosystems). All miRs assay primers used in this study were purchased commercially (Ribobio). Primer efficiencies were determined by a standard curve. The relative miRNAs’ expressions were calculated by the efficiency-corrected ΔΔCt method and normalized to the exogenous control cel-miR-39-3p [19]. Each sample was analyzed in duplicate, and the mean was used to determine the miRNA levels.

### Statistical analysis

All continuous parametric values were presented as the mean ± SEM, as determined from at least three independent experiments. Statistical significance was assessed using a one-way ANOVA. P < 0.05 was considered statistically significant. The statistical analysis was conducted using SPSS 19.0 software (SPSS Software, Chicago, IL, USA).

Because some of the six miRNA expressions were related to the clinical pregnancy outcomes of IVF-ET and RM pathogenesis, we analyzed the ability of the combination of the six miRNA expressions in plasma and serum to predict the clinical pregnancy outcome for IVF-ET and potential diagnostic biomarkers for RM using receiver operating characteristic (ROC) curves and calculating the AUC with the 95% confidence intervals after a logistic regression. The sensitivity and specificity of the optimal cut-off were calculated. The statistical tests were performed using Stata 15.0 (StataCorp, TX, USA). The results were considered significant when P < 0.05.

## Results

### Clinical characteristics of enrolled participants

In total, 16 RM patients who had experienced at least two consecutive spontaneous early miscarriages before the 12th gestational week were recruited, and information about their personal history of thromboembolic diseases and previous early pregnancy losses were inquired. Meanwhile, 29 matched NP women were concurrently enrolled. No significant differences in the average age and gestational week at sampling were observed between RM patients and NP women (Table 1).

**Table 1:** Clinical characteristics of the recruited recurrent miscarriage (RM) patients (n = 16) and normal pregnant (NP) women (n = 29)

Clinical characteristics	RM (mean ± SD)	NP (mean ± SD)	*P* value
Age (years)	29.9 ± 0.84	28.0 ± 0.92	0.208
Gestational week	8.70 ± 0.32	8.13 ± 0.15	0.113
Pregnant history	3.06 ± 1.75	3.00 ± 1.83	0.922
Childbearing history	0.05 ± 0.24	0.83 ± 0.85	0.001
Miscarriage history	2.94 ± 1.78	0.00 ± 0.00	0.000

For the IVF-ET cohort, a total of 146 women undergoing IVF-ET were enrolled. By the end of the first trimester, 72 women were diagnosed by imaging with ultrasonography as clinically pregnant (Pregnancy), whereas another 74 women were diagnosed as non-pregnant after the ET (Failure). Fifty women in the Pregnancy group and 53 participants in the Failure group have never received the ET treatment before, and 22 women in the Pregnancy group and 21 participants in the Failure group had received ET treatment at least once. No significant differences in the average age, number of transferred embryos, infertility duration, BMI, or peripheral blood concentrations of FSH and LH were observed between the Pregnancy and Failure groups (Table 2).

**Table 2:** Clinical characteristics of the recruited women undergoing IVF-ET treatment

History of embryo transfer treatment	Clinical characteristics	Outcome of embryo transfer				*P* value
		Failure		Pregnancy		
		n	Mean ± SD	n	Mean ± SD	
1st time	Age (years)	53	33.8 ± 0.73	50	31.0 ± 0.57	0.0040
	Number of transferred embryo		1.98 ± 0.6312		1.98 ± 0.3488	0.9788
	Infertility duration (years)		5.74 ± 0.69		4.89 ± 0.50	0.3234
	BMI (kg/m^2^)		22.1 ± 0.48		21.9 ± 0.32	0.7683
	FSH (mIU/ml)		7.49 ± 0.43		8.14 ± 0.43	0.2831
	LH (mIU/ml)		3.85 ± 0.32		4.37 ± 0.44	0.3418
2nd–4th time	Age (years)	21	31.2 ± 0.77	22	31.6 ± 1.15	0.8275
	Number of transferred embryo		1.95 ± 0.47		1.86 ± 0.99	0.4334
	Infertility duration (years)		3.95 ± 0.63		5.91 ± 0.82	0.0670
	BMI (kg/m^2^)		21.4 ± 0.51		22.4 ± 0.50	0.1875
	FSH (mIU/ml)		6.74 ± 0.41		6.45 ± 0.38	0.6063
	LH (mIU/ml)		3.92 ± 0.68		5.32 ± 0.78	0.1864
Total	Age (years)	74	31.0 ± 0.59	72	32.5 ± 0.58	0.6326
	Number of transferred embryo		1.99 ± 0.45		1.93 ± 0.46	0.3360
	Infertility duration (years)		4.93 ± 0.43		5.41 ± 0.54	0.4898
	BMI (kg/m^2^)		22.0 ± 0.29		22.0 ± 0.36	0.9880
	FSH (mIU/ml)		7.50 ± 0.37		7.50 ± 0.31	0.9936
	LH (mIU/ml)		3.95 ± 0.36		4.44 ± 0.32	0.2989

### Validation of differential villus miR expressions

**Fig. 1:**
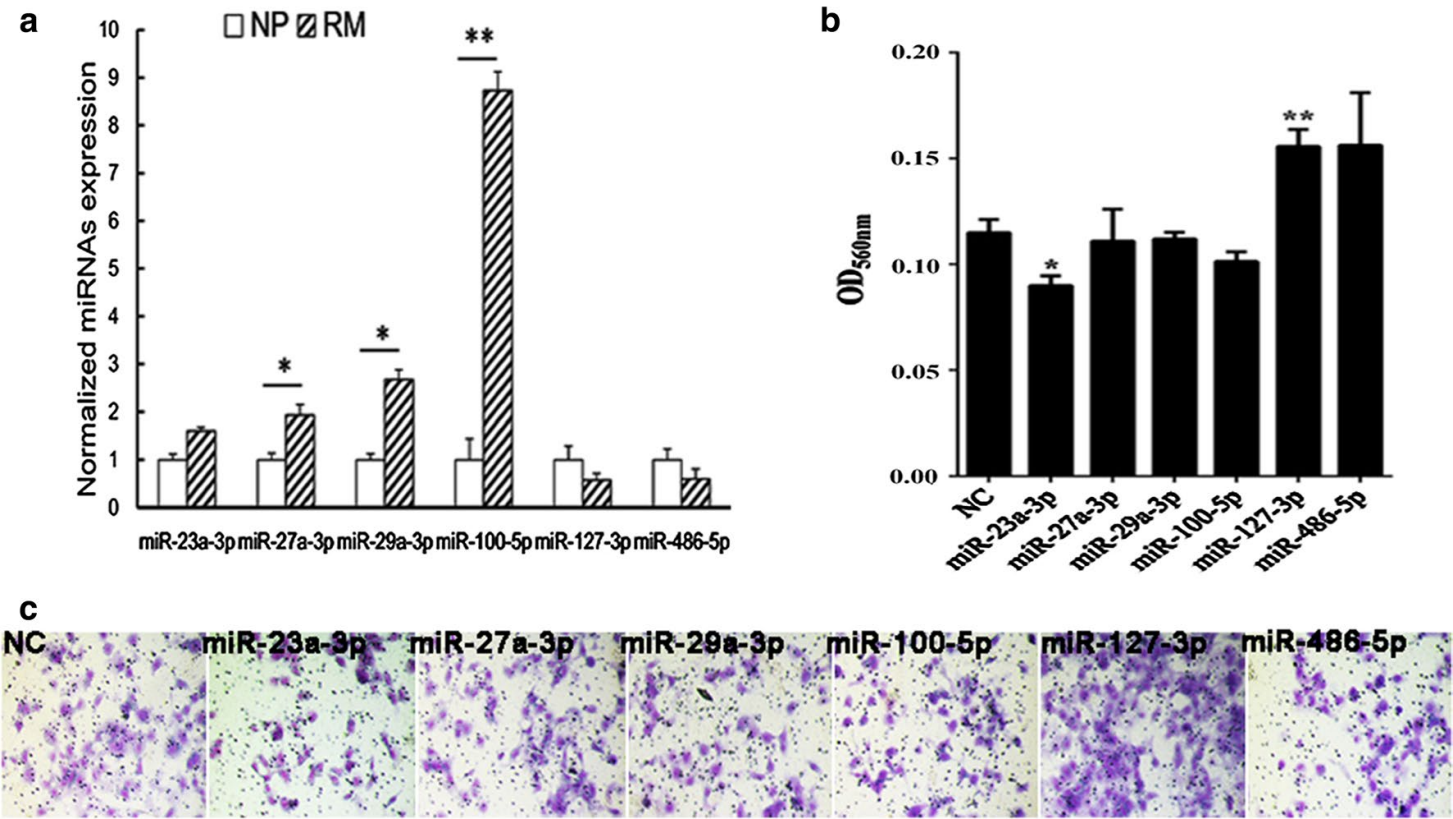
**a** Comparison of the villus expression levels of miR-23a-3p, miR-27a-3p, miR-29a-3p, miR-100-5p, miR-127-3p and miR-486-5p between RM patients and NP (n = 5) by real-time quantitative PCR analysis. *(P < 0.05) and **(P < 0.01) indicate significant differences. **b** Quantifications of HTR8/SVneo cell invasiveness by the Transwell assay. *NC* transfected by NC. MiR-23a-3p, miR-27a-3p, miR-29a-3p, miR-100-5p, miR-127-3p and miR-486-5p, transfected by the corresponding mimic of each miRNA. This experiment was repeated three times, and a triple-well was set up for each group at each time (n = 3 × 3) (*P < 0.05; **P < 0.01; vs NC). **c** Representative images of filters containing cells from the Transwell assay. NC, transfected by the negative control sequence of miRNA mimics; miR-23a-3p, miR-27a-3p, miR-29a-3p, miR-100-5p, miR-127-3p and miR-486-5p, transfected by the corresponding mimic of each miRNA

In our previous study, the differential villus expression of miR-100-5p in RM patients was validated by qPCR [13]. MiR-23a-3p, miR-27a-3p, miR-29a-3p and miR-127-3p were also screened out by deep-sequencing, but they were not listed as RM-related differentially villus expressed miRs in our published data because their fold changes were less than 2.0 (Additional file 1: Table S1). In this study, we validated the differential villus expression of miR-23a-3p, miR-27a-3p, miR-29a-3p, miR-127-3p and miR-486-5p and that of miR-100-5p (as a positive control) by qPCR. As expected, the villus expressions of miR-27a-3p, miR-29a-3p and miR-100-5p were significantly up-regulated in RM patients, and the villus expression of miR-23a-3p in RM patients also showed an increasing tendency, whereas the expressions of miR-127-3p and miR-486-5p were decreased in RM patients, but no significant differences were observed (Fig. 1a).

### Villus and decidual expression localization of miRs

**Fig. 2:**
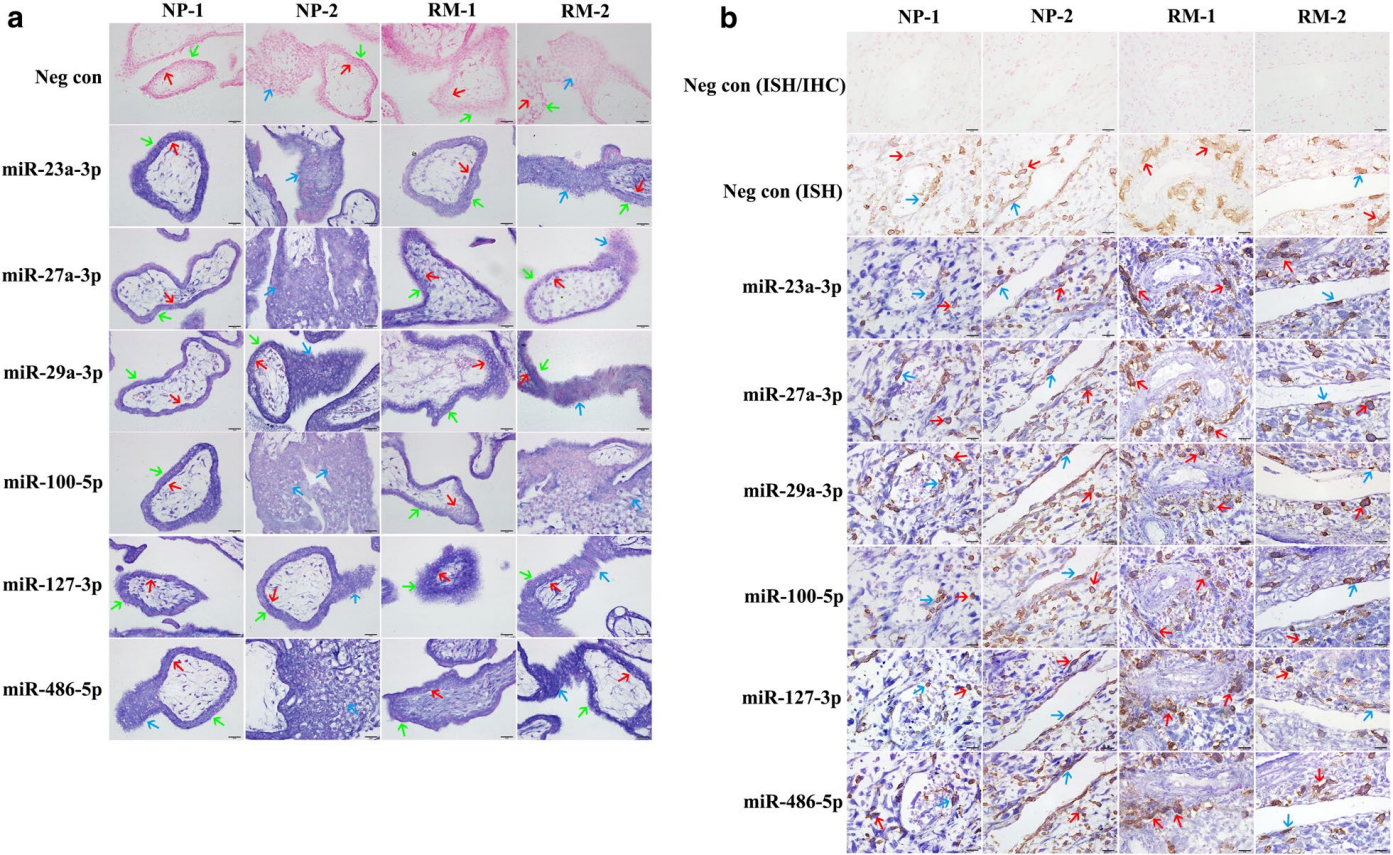
Villus (**a**) and decidual (**b**) expression localization of miR-23a-3p, miR-27a-3p, miR-29a-3p, miR-100-5p, miR-127-3p and miR-486-5p (blue signals) in normal pregnant women (NP1 and NP2) and recurrent miscarriage patients (RM1 and RM2) by in situ hybridization analysis. A scramble miRNA probe was used as a negative control (Neg con). In the villus tissues, the red arrow represents cytotrophoblast cells, the green arrow represents syncytiotrophoblast cells, and the blue arrow represents column cytotrophoblast cells. In decidual tissues, HLA-G protein (brown signals) was detected by immunohistochemistry to show extravillous trophoblast cells invaded in decidua, the red arrow represents interstitial trophoblast cells, and the blue arrow represents endovascular trophoblast cells. Scale bar = 20 μm

In situ hybridization analyses were performed to localize the expressions of miRs using 2 paired samples of RM patients (RM1 and RM2) and NP women (NP1 and NP2) (Additional file 1: Table S3). HLA-G protein signals were detected as the biomarker of extravillous trophoblast cells by an immunohistochemistry analysis to confirm whether the decidual tissues were from implantation sites. The results showed that positive signals of these miRs were detected in the villi of early pregnancy and were widely localized in cytotrophoblast cells, syncytiotrophoblasts and column cytotrophoblasts (Fig. 2a). In decidual tissues, the positive miRNA signals were observed not only in maternal decidual stromal cells but also in embryonic interstitial trophoblast cells and endovascular trophoblast cells (Fig. 2b), indicating that the expressions of these miRs were not villus tissue-specific at the maternal–fetal interface during early pregnancy.

### Effects of miRs on HTR8/SVneo cell invasion

The results of the Transwell assay showed that the migrated cell number of miR-23a-3p mimic-transfected HTR8/SVneo cells was significantly reduced, whereas that of miR-127-3p mimic-transfected cells was significantly increased compared to the corresponding control treated cells (Fig. 1b, c). This result indicates an inhibitory effect of miR-23a-3p and a stimulating effect of miR-127-3p on the cell invasiveness in vitro. However, there was no significant difference in the migrated cell number of miRNA inhibitor-transfected HTR8/SVneo cells (Additional file 1: Fig. S1).

### Differential levels of miRs in the peripheral blood of RM patients

**Fig. 3:**
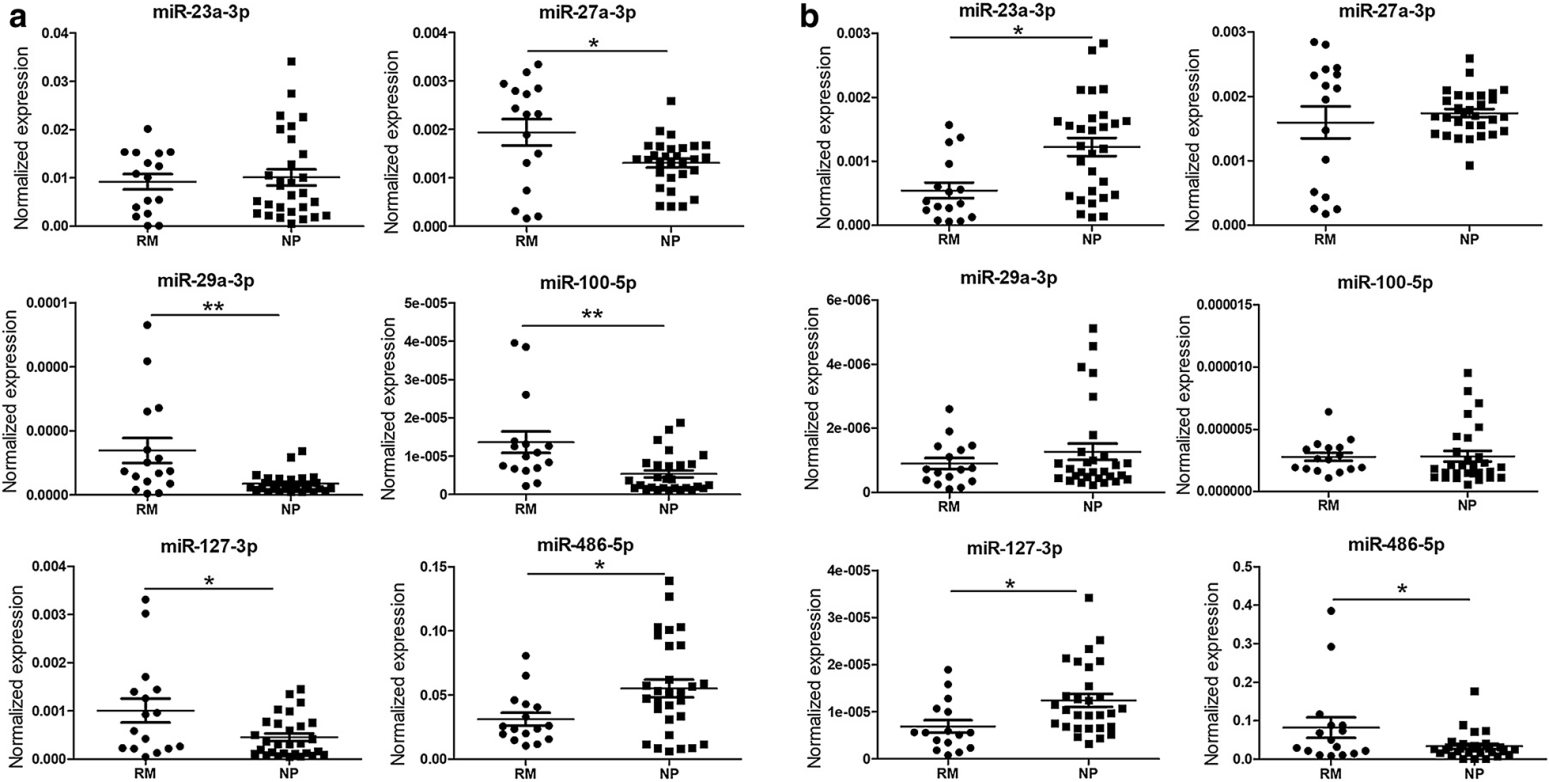
Comparison of the relative levels of miR-23a-3p, miR-27a-3p, miR-29a-3p, miR-100-5p, miR-127-3p and miR-486-5p in plasma (**a**) and serum (**b**) of peripheral blood between RM patients and NP controls. *Significantly different from control (P < 0.05); **significantly different from control (P < 0.01). *NP* normal pregnant women, *RM* recurrent miscarriage patients

Peripheral blood samples were obtained from 16 RM patients and 29 NP women (Table 1), and the results of the qPCR analysis showed that compared to NP women, the plasma levels of miR-27a-3p, miR-29a-3p, miR-100-5p and miR-127-3p were significantly elevated, whereas the level of miR‑486-5p was remarkably decreased in RM patients, but no significant differences in plasma miR-23a-3p levels were reported (Fig. 3a). In serum, the levels of miR-23a-3p and miR-127-3p were decreased, whereas the level of miR-486-5p was elevated in RM patients compared to NP women; however, no significant differences in the serum levels of miR-27a-3p, miR-29a-3p and miR-100-5p were detected (Fig. 3b).

### Predictive value of miRs for the potential diagnostic biomarkers of RM

**Fig. 4:**
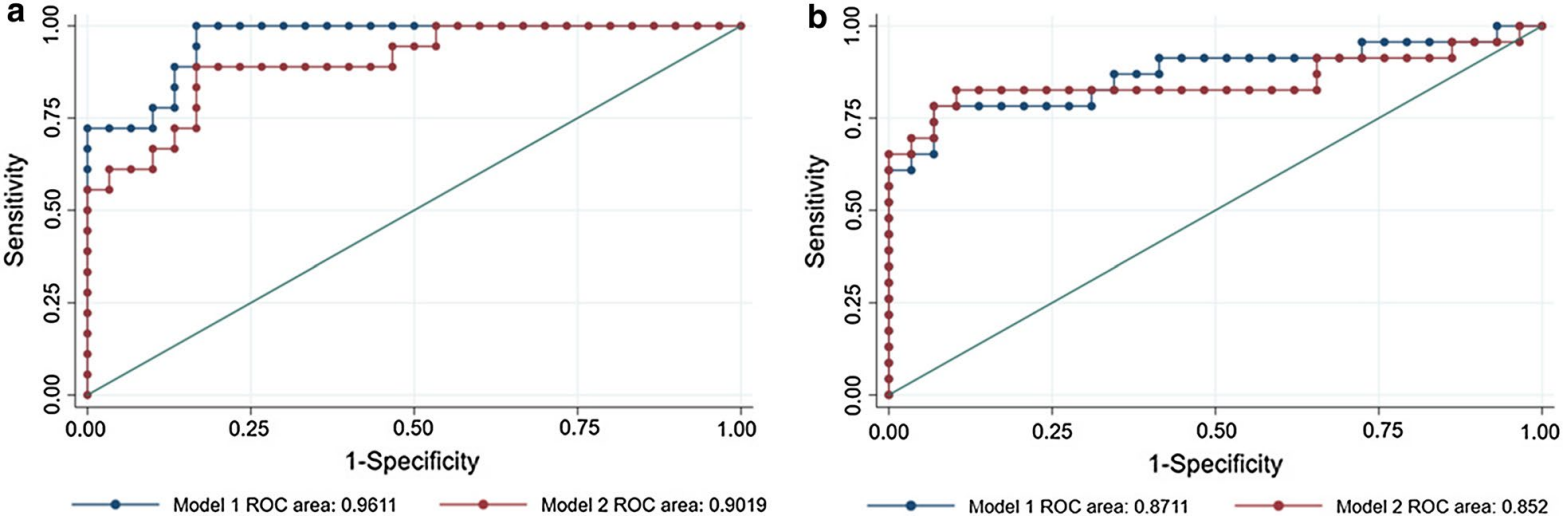
Predictive value assessment by ROC analysis in the plasma and serum of RM patients. **a** ROC curve of miRs for peripheral blood plasma. Curves of Model 1 (combination of all 6 miRs: miR-23a-3p, miR-27a-3p, miR-29a-3p, miR-100-5p, miR-127-3p and miR-486-5p) and Model 2 (combination of 2 miRs: miR-127a-3p and miR-486-5p) for peripheral blood plasma; **b** ROC curves of miRs (combined 6 miRs) for peripheral blood serum. Curves of Model 1 (combination of all 6 miRs: miR-23a-3p, miR-27a-3p, miR-29a-3p, miR-100-5p, miR-127-3p and miR-486-5p) and Model 2 (combination of 3 miRs: miR-23a-3p, miR-29a-3p and miR-486-5p) for peripheral blood serum

To appraise the predictive value of these circulating miRs as potential diagnostic biomarkers of RM, the levels of miRs (miR-23a-3p, miR-27a-3p, miR-29a-3p, miR-100-5p, miR-127-3p and miR-486-5p) in peripheral blood plasma or serum were calculated using logistic models. The ROC analysis for miRs in plasma indicated that the performance of Model 1 (combination of all six miRs) and Model 2 (combination of only miR-127a-3p and miR-486-5p) for predicting RM reached 0.96 (0.92–1.00) and 0.90 (0.81–0.99), respectively. Model 1 has a sensitivity of 100% and a specificity of 83.3% with 89.6% correctly classified, whereas Model 2 has a sensitivity of 88.9% and a specificity of 80.0% with 83.3% correctly classified. These preliminary results suggest that the plasma levels of miR-127a-3p and miR-486-5p could serve as predictive factors for RM (Table 3 and Fig. 4a).

**Table 3:** Receiver operating characteristic analysis in potential diagnostic biomarkers of RM in plasma

Roc index	Prediction for clinical pregnancy outcome	
	Model 1^a^ (combination of the six miRNAs)	Model 2^b^ (combination of miR-127a-3p and miR-486-5p)
AuROC (95% CI)*	0.96 (0.92, 1.00)	0.90 (0.81, 0.99)
Sensitivity (%)	100	88.9
Specificity (%)	83.3	80.0
Positive predictive value (%)	78.3	72.7
Negative predictive value (%)	100	92.3
Correctly classified (%)	89.6	83.3
Cut-off value	0.213^a^	0.336^b^

* No significant difference in ROC area between Model 1 and Model 2

^a^Model 1, Cut-off value = 0.213, (miR-23a-3p, miR-27a-3p, miR-29a-3p, miR-100-5p, miR-127a-3p, miR-486-5p) (6.312687, 6.228964, 13.70655, 13.10668, 9.521886, 4.800646)

^b^Model 2, Cut-off value = 0.336, (miR-100-5p, miR-486-5p) (13.27899, 4.726676)

The ROC analysis for miRs in serum indicated that the performance of Model 1 (combination of all six miRs) and Model 2 (combination of miR-23a-3p, miR-29a-3p and miR-486-5p) for predicting RM reached 0.87 (0.76–0.98) and 0.85 (0.72–0.98), respectively. Model l has a sensitivity of 78.3% and a specificity of 93.1% with 86.5% correctly classified, whereas Model 2 has a sensitivity of 82.1% and a specificity of 89.7% with 86.5% correctly classified. This indicated that the serum levels of miR-23a-3p, miR-29a-3p and miR-486-5p could also serve as predictive factors for RM (Table 4 and Fig. 4b).

**Table 4:** Receiver operating characteristic analysis in potential diagnostic biomarkers of RM in serum

Roc index	Prediction for clinical pregnancy outcome	
	Model 1^a^ (combination of the six miRNAs)	Model 2^b^ (combination of miR-23a-3p, miR-29a-3p and miR-486-5p)
AuROC (95% CI)*	0.87 (0.76, 0.98)	0.85 (0.72, 0.98)
Sensitivity (%)	78.3	82.1
Specificity (%)	93.1	89.7
Positive predictive value (%)	90.0	86.4
Negative predictive value (%)	84.4	86.7
Correctly classified (%)	86.5	86.5
Cut-off value	0.396^a^	0.382^b^

* No significant difference in ROC area between Model 1 and Model 2

^a^Model 1, Cut-off value = 0.396, (miR-23a-3p, miR-27a-3p, miR-29a-3p, miR-100-5p, miR-127a-3p, miR-486-5p) (8.986689, 6.043169, 14.62186, 13.73859, 12.26344, 6.515767)

^b^Model 2, Cut-off value = 0.382, (miR-23a-3p, miR-29a-3p, miR-48s5p) (5.41586, 14.80947, 2.419319)

### Association of the peripheral blood levels of miRs with the outcomes of IVF-ET

**Fig. 5:**
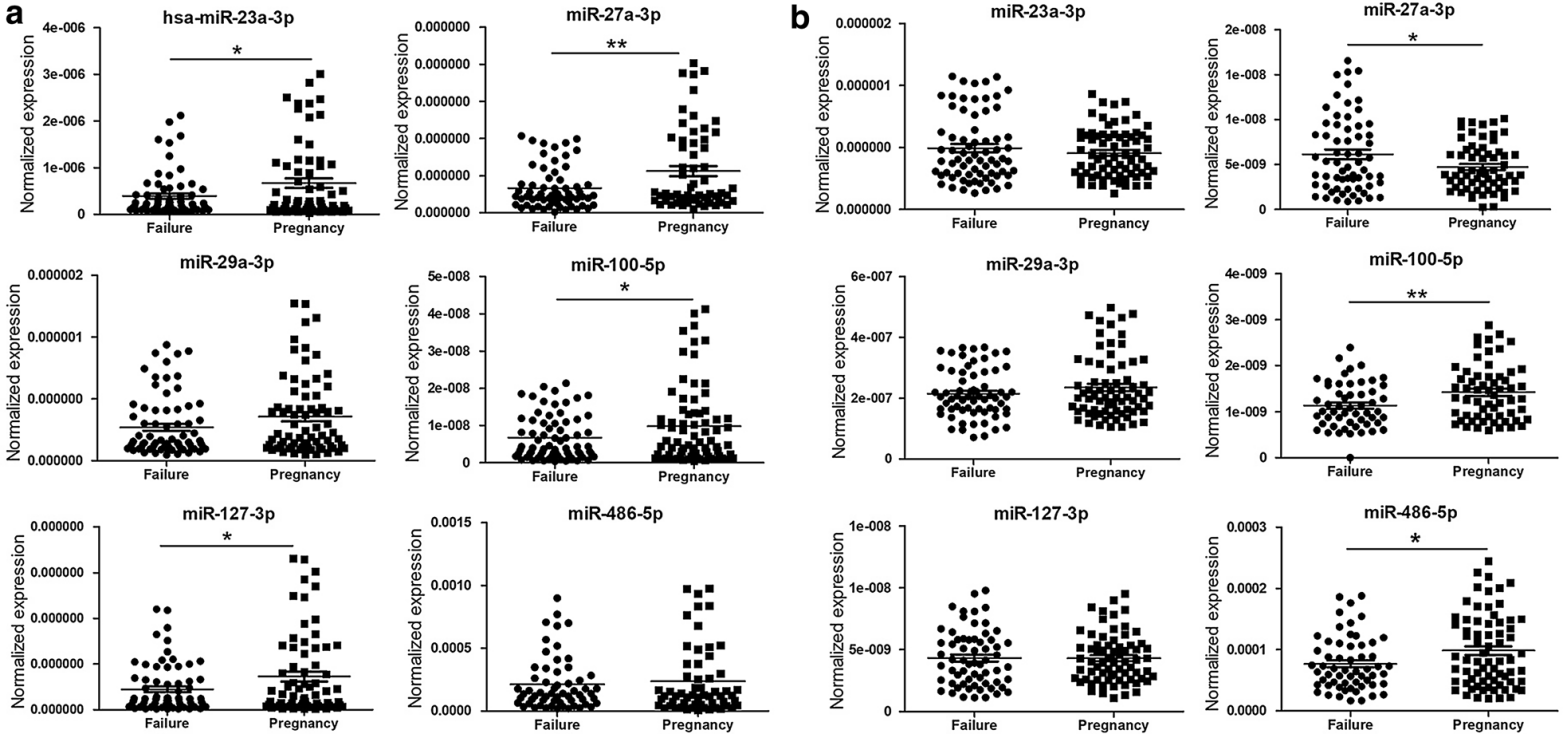
Comparison of the relative levels of miR-23a-3p, miR-27a-3p, miR-29a-3p, miR-100-5p, miR-127-3p and miR-486-5p in the plasma (**a**) and serum (**b**) of peripheral blood between pregnant women (n = 72) and non-pregnant women (n = 74) after embryo transfer in IVF-ET cycle. *Significantly different from control (P < 0.05); **significantly different from control (P < 0.01). Failure, non-pregnant women after ET treatment; Pregnancy, pregnant women after ET treatment

To preliminarily evaluate the potential of these circulating miRs to be used as clinical indexes to predict the outcome of IVF-ET, the plasma and serum levels of miR-23a-3p, miR-27a-3p, miR-29a-3p, miR-100-5p, miR-127-3p and miR-486-5p were compared between 72 pregnant and 74 non-pregnant women after ET treatment during an IVF cycle. The results showed that compared to pregnant women, the plasma levels of miR-23a-3p, miR-27a-3p, miR-100-5p and miR-127-3p were significantly decreased in non-pregnant women (Fig. 5a), and the serum level of miR‑27a-3p was significantly elevated, whereas the levels of miR-100-5p and miR-486-5p were remarkably decreased in non-pregnant women (Fig. 5b). We also attempted to take the history of ET treatment into account; however, there was no significant difference in the plasma (Additional file 1: Figs. S2A, S3A) or serum (Additional file 1: Figs. S2B, S3B) levels of any miRs.

### Predictive value of miRs for the outcome of IVF-ET

To appraise the predictive value of these circulating miRs for the clinical outcome of IVF-ET, the levels of miRs (miR-23a-3p, miR-27a-3p, miR-29a-3p, miR-100-5p, miR-127-3p and miR-486-5p) in peripheral blood plasma or serum were calculated using logistic models. The plasma levels of miR-127a-3p and miR-486-5p were shown to significantly predict the outcome of IVF-ET; however, in serum, none of the selected miRs could significantly predict the outcome (Additional file 1: Table S4). The ROC analysis for miRs in plasma indicated that the performance of both Model 1 (combination of all six miRs) and Model 2 (combination of miR-127a-3p and miR-486-5p) for predicting clinical pregnancy reached 0.62 (0.53–0.71). Model 1 has a sensitivity of 68.1% and a specificity of 54.1% with 61% correctly classified, whereas Model 2 has a sensitivity of 50% and a specificity of 75.3% with 62.3% correctly classified (Table 5 and Additional file 1: Fig. S4A). The ROC curve analysis for miRNAs in serum indicated that the performance of the combination of the six miRs for predicting clinical pregnancy reached 0.53 (0.44–0.61) with a sensitivity of 27.8% and a specificity of 85.1% (Additional file 1: Table S5, Fig. S4B).

**Table 5:** Receiver operating characteristic analysis in prediction for outcome of IVF-ET by the selected miRs in peripheral blood plasma

Roc index	Prediction for clinical pregnancy outcome	
	Model 1^a^ (combination of the six miRs)	Model 2^b^ (combination of miR-127a-3p and miR-486-5p)
AuROC (95% CI)*	0.62 (0.53, 0.71)	0.62 (0.53, 0.71)
Sensitivity (%)	68.1	50.0
Specificity (%)	54.1	75.3
Positive predictive value (%)	59.0	65.5
Negative predictive value (%)	63.5	60.4
Correctly classified (%)	61.0	62.3
Cut-off value	0.469^a^	0.533^b^

* No significant difference in ROC area between Model 1 and Model 2

^a^Model 1: Cut-off value = 0.469; (miR-23a-3p, 22.112194); (miR-27a-3p, 28.546087); (miR-29a-3p, 23.40597); (miR-100-5p, 28.019193); (miR-127-3p, 27.498337); (miR-486-5p, 12.804071)

^b^Model 2: Cut-off value = 0.533; (miR-127-3p, 28.653163); (miR-486-5p, 14.214297)

Furthermore, we took the history of IVF-ET treatment into account to evaluate the prediction for clinical pregnancy outcomes of IVF-ET by the six miRNAs in plasma and serum after fitting logistic models. When adjusting for the number of transplantation treatments, the ROC curve analysis for miRNAs in plasma indicated that the performance of the full model (combination of all six miRNAs) and the reduced model (combination of miR-127a-3p and miR-486-5p) for predicting clinical pregnancy reached 0.61 (0.52–0.71). The full model has a sensitivity of 68.1% and a specificity of 51.4% with 59.9% correctly classified, and the reduced model has a sensitivity of 50% and a specificity of 70% with 59.9% correctly classified (Additional file 1: Table S6).

The ROC curve analysis for miRNAs in serum indicated that the performance of the combination of all six miRNAs for predicting clinical pregnancy reached 0.52 (0.42–0.61) with a sensitivity of 12.5% and a specificity of 97.1% (Additional file 1: Table S7). No expression level of miRNAs was shown to be able to significantly predict the clinical pregnancy outcome in either plasma or serum when stratified according to a history of IVF-ET treatment.

## Discussion

The present exploratory study validated the differential villus expressions of miR-23a-3p, miR-27a-3p, miR-29a-3p, miR-100-5p, miR-127-3p and miR-486-5p in RM patients compared to NP women and localized their expression in the villus and decidual tissues of early pregnancy. The expression profiles of these miRs were also investigated in the peripheral blood of RM patients and women undergoing the IVF-ET procedure, and the predictive value of these miRs for the outcome of IVF-ET was calculated preliminarily.

We demonstrated that, consistent with the previous results of deep-sequencing analysis, the villus expression levels of miR-27a-3p, miR-29a-3p and miR-100-5p were significantly up-regulated in RM patients compared to NP women, and an up-regulation trend of miR-23a-3p expression, in addition to down-regulated trends of miR-127-3p and miR-486-5p expressions, were observed, although no significant difference was counted. The expressions of these six miRs were localized both in maternal DSCs and fetal trophoblast cells.

miR-23a has been shown to promote trophoblast cell apoptosis and its expression level is up-regulated in the placenta of PE patients [20], but the role of miR-23a-3p in RM patients has not yet been revealed. Given that miR-23-3p inhibits type II collagen expression in chondrocytes [21] and that the increased expression of type II collagen can promote cell migration [22], miR-23a-3p might suppress trophoblast cell invasion. Coincidentally, the inhibitory effect of miR-23a-3p over-expression on HTR8/SVneo cell invasiveness was observed in this study.

The association between miR-27a polymorphisms and RM was recently identified [23]. Here, a significantly increased villus expression of miR-27a-3p in RM patients was also validated. However, although miR-27a-3p over-expression promotes the migration and invasion of hepatocellular carcinoma [24] and nasopharyngeal carcinoma cells [25], no obvious effect of miR-27a-3p over-expression on the invasiveness of HTR8/SVneo cells was observed here. Very recently, it was reported that miR-27a-3p over-expression in human granulosa-like tumor cells inhibits cell proliferation and promotes cell apoptosis [26]. Thus, ectopic miR-27a-3p in placental villi might dysregulate the proliferation and apoptosis of trophoblast cells, resulting in pregnancy loss.

The expression of miR-29a at the placental creta sites is evidently lower compared to non-creta sites, and its over-expression induces HTR8/SVneo cell apoptosis [27]. Thus, it is reasonable for us to speculate that the significantly increased expression of miR-29a-3p in the placenta villi of RM patients observed in this study might be involved in the RM pathogenesis by leading to excessive trophoblast cell apoptosis during early pregnancy.

The role of miR-100-5p in reproduction remains mostly unknown, but the stimulating effect of miR-100-5p over-expression on neuronal cell apoptosis was reported [28]. Herewith, the villus miR-100-5p expression was detected to be significantly up-regulated in RM patients; thus, we supposed that similar to miR-29-3p, the dysregulation of miR-100-5p expression might also disturb trophoblast cell apoptosis, resulting in early pregnancy loss.

MiR-127-3p was reported to consistently induce the migration and invasion of glioblastoma cells [29]; this study showed that HTR8/SVneo cell invasive activity was effectively promoted by miR-127-3p over-expression. The decreasing trend of its villus expression in RM patients suggested that it might be involved in RM pathogenesis by reducing trophoblast cell invasion.

MiR-486-5p was another miRNA that showed a decreasing trend of villus expression in RM patients, and its lower expression level in the cumulus cells of polycystic ovary syndrome patients was also reported [30]. In lung cancer cells, miR-486-5p over-expression could inhibit cell proliferation and migration, but it induced cell apoptosis [31], indicating that insufficient functions of miR-486-5p might lead to implantation failure by the dysregulation of trophoblast cell activities.

We also observed the effect of these miRNA inhibitors on cell invasive activities, but no significant differences were identified. Because there are several important requirements for an miRNA inhibitor to achieve effective downregulation of a targeted miRNA, such as stability, specificity and affinity [32, 33], further studies must be performed using synthetic inhibitors with high efficacy or transfection with lentiviral plasmid vector.

Given that circulating miRs could reflect pathological status and be detectable in peripheral blood [34] and that several differentially expressed miRs in the placenta of complicated pregnancies could be detected in maternal peripheral blood [35], we speculated that these six miRs might also represent various peripheral blood levels between RM patients and NP women. In addition, although both blood plasma and serum are commonly used to detect the circulating miRs, it has been suggested that serum is better than plasma for use in research because hemolysis can influence the circulating miR abundance [36]; we thus determined the plasma and serum levels of six detected miRs in this study.

Encouragingly, the results showed that compared to NP women, the plasma levels of miR-27a-3p, miR-29a-3p, miR-100-5p and miR-127-3p in RM patients were significantly increased, whereas the plasma miR-486-5p level was remarkably decreased. By contrast, the serum levels of miR-23a-3p and miR-127-3p were significantly lower, whereas the serum miR-486-5p level was remarkably higher. The higher plasma levels of miR-27a-3p, miR-29a-3p and miR-100-5p, and the lower level of miR-486-5p, were in consistent with their differential villus expression levels. An opposite relationship between plasma levels and serum levels of miR-23a-3p, miR-127-3p and miR-486-5p was observed, and we thought this might be caused by different resources of these miRs.

More interestingly, miR-27a and miR-29a were reported to be potential non-invasive diagnostic or predictive biomarkers of PE because increased levels of these miRs were observed both in the plasma and placenta of PE patients [15, 16]. Moreover, an elevated serum miR-127 level was also demonstrated to be associated with small-for-gestational-age [17], making our observations more convincing, and indicating that miR-27a-3p, miR-29a-3p, miR-100-5p, miR-127-3p and miR-486-5p are promising potential diagnostic biomarkers for pathologic pregnancies, including RM, PE and IUGR. To appraise the predictive value of these circulating miRs as potential diagnostic biomarkers of RM, the levels of 6 miRs in the peripheral blood plasma or serum were calculated using logistic models. The results showed that miR-127a-3p and miR-486-5p could serve as predictive factors for RM in plasma, and iR-23a-3p, miR-29a-3p and miR-486-5p could serve as predictive factors for RM in serum. The difference in the types of miRNAs between the plasma and serum may be indicative of the difference in the confined location of miRNAs in peripheral blood. The potential of circulating miRNAs as blood-based biomarkers for RM is promising. Importantly, it was expected that combining multiple miRNAs into the miRNA profile may provide greater accuracy than can be expected from the assessment of a single miRNA [37].

Furthermore, given that implantation failure after IVF-ET treatment has been a limiting factor for the improvement of the IVF success rate and that miR-29a has been explored as a potential predictive biomarker for outcomes of the IVF process [38], we also observed an association between the peripheral blood levels of these six miRs and the outcomes of embryo transfer in an IVF cycle. The distinctly lower plasma levels of miR-23a-3p, miR-27a-3p, miR-100-5p and miR-127-3p, and the serum levels of miR-100-5p and miR-486-5p, in addition to the remarkably higher serum level of miR-27a-3p, were observed to be correlated with the failure of ET, showing an almost inverse change compared to the RM cohort. This could be explained by the different sampling time. Samples from the RM cohort were collected after the implantation failure, of which fetal factors were included, whereas samples of the IVF-ET cohort were collect prior to embryo transfer and lacked fetal factors. Alterations in peripheral blood levels of these six circulating miRs are summarized in Table 6.

**Table 6:** Alterations in peripheral blood levels of miRs

	miR levels of RM patients (vs NP women)						miR levels of non-pregnant women in IVF-ET (vs pregnant women)			
	Villi		Plasma		Serum		Plasma		Serum	
	FC	P	FC	P	FC	P	FC	P	FC	P
miR-23a-3p	1.602	0.322	− 1.097	0.726	− 2.252	*0.002*	− 1.704	*0.021*	1.086	0.326
miR-27a-3p	1.929	*0.045*	1.485	*0.010*	− 1.090	0.470	− 1.691	*0.003*	1.305	*0.027*
miR-29a-3p	2.676	*0.031*	3.926	*0.001*	− 1.412	0.321	− 1.316	0.069	− 1.100	0.202
miR-100-5p	9.732	*0.000*	2.553	*0.001*	− 1.014	0.948	− 1.462	*0.033*	− 1.261	*0.007*
miR-127-3p	− 1.740	0.105	2.227	*0.011*	− 1.807	*0.011*	− 1.620	*0.027*	1.001	0.989
miR-486-5p	− 1.663	0.220	− 1.763	*0.021*	2.455	*0.029*	− 1.113	0.593	− 1.284	*0.023*

In RM and NP group: Positive fold change (FC) means up-regulated in RM patients and negative fold change means down regulated in RM patients compared to NP women. In IVF-ET group: Positive fold change (FC) means up-regulated in non-pregnant women in IVF-ET and negative fold change means down regulated in non-pregnant women in IVF-ET compared to pregnant women. Results were considered significant when P < 0.05

In particular, the combination of plasma levels of these six miRs discriminated the outcome of IVF-ET with a sensitivity of 68.1% and a specificity of 54.1%, whereas the combination of plasma miR-127-3p and miR-486-5p levels showed a slightly lower sensitivity (50.0%) but a notable higher specificity (75.3%), providing potential biomarkers to efficiently predict the outcomes of IVF-ET treatment. When the IVF-ET participants were further sub-grouped according to the history of embryo transfer treatment and the prediction for clinical pregnancy outcomes of IVF-ET by the six miRNAs in plasma and serum was determined after fitting logistic models, no miRNA expression levels were able to predict the clinical pregnancy outcome significantly in both plasma and serum. This might be due to the limited number of IVF-ET patients when sub-grouped according to the history of embryo transfer treatment. We should increase the case number to strength our conclusion in future investigations.

## Conclusions

This exploratory study suggested that peripheral blood levels of circulating miR-23a-3p, miR-27a-3p, miR-29a-3p, miR-100-5p, miR-127-3p and miR-486-5 were associated with both RM and the outcomes of embryo transfer to varying degrees, thereby presenting potential diagnostic or predictive biomarkers for RM and the outcomes of IVF-ET treatment. Further validation of these preliminary findings might lead to the development of novel non-invasive diagnostic and predictive means for the improvement of clinical infertility management. There were several limitations in this study. The low abundance of these circulating miRs, inadequate sample sizes, and potentially insufficient utilization of available data should influence the confidence level of this study, calling for a large-scale multicenter trial in the future.

## Additional file

**Additional file 1.** Additional figures and tables.

## Abbreviations

RM: recurrent miscarriage
MicroRNAs: miRs
IVF-ET: in vitro fertilization-embryo transfer
