# Supplementary material for: Association of the peripheral blood levels of circulating microRNAs with both recurrent miscarriage and the outcomes of embryo transfer in an in vitro fertilization process
Source: J Transl Med. 2018 Jul 4;16:186. doi: 10.1186/s12967-018-1556-x (PMC6032771; doi:10.1186/s12967-018-1556-x)
Supplement: Supplementary file 1 — Additional file 1. Additional figures and tables. [file 12967_2018_1556_MOESM1_ESM.docx]

**FIGURE S1**

(A) Quantifications of HTR8/SVneo cell invasiveness by transwell assay. NC, transfected by NC. MiR-23a-3p, miR-27a-3p, miR-29a-3p, miR-100-5p, miR-127-3p and miR-486-5p, transfected by the corresponding inhibitors of each miRNAs. This experiment was repeated three times, and triple-well was set up for each group in each time (n=3×3). (B) Representative images of filters containing cells from the transwell assay. NC, transfected by negative control sequence of miRNA inhibitors; miR-23a-3p, miR-27a-3p, miR-29a-3p, miR-100-5p, miR-127-3p and miR-486-5p, transfected by the corresponding inhibitors of each miRNAs.

**
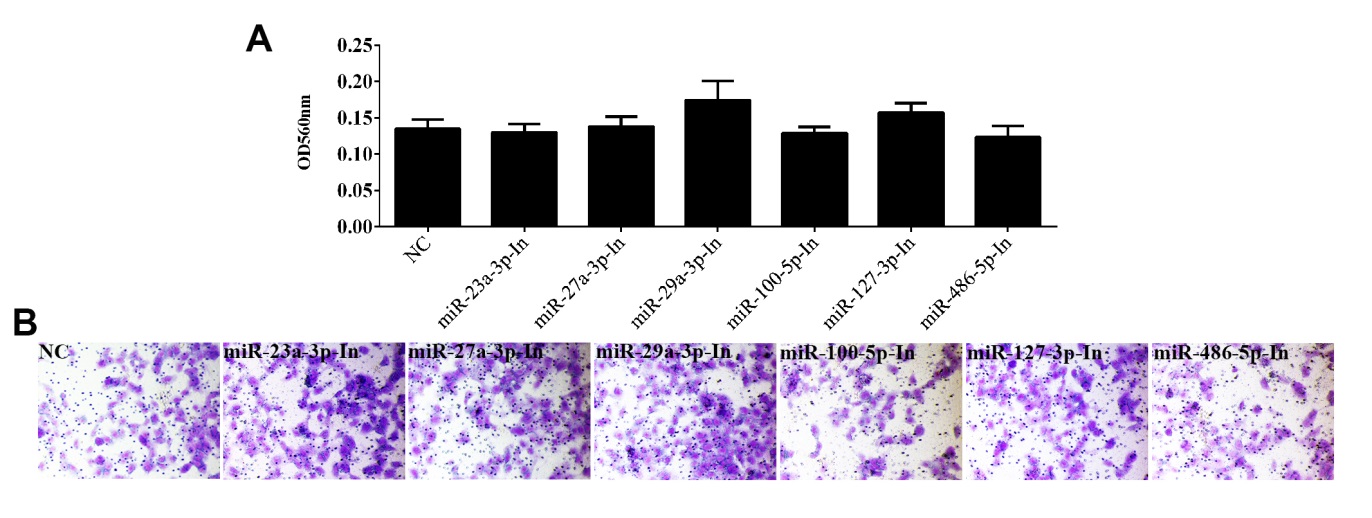
**

**FIGURE S2**

Comparison of relative levels of miR-23a-3p, miR-27a-3p, miR-29a-3p, miR-100-5p, miR-127-3p and miR-486-5p in plasma (A) and serum (B) of peripheral blood between pregnant women (n=50) and non-pregnant women (n=53) after the first-time embryo transfer in IVF-ET cycle. Failure, non-pregnant women after ET treatment; Pregnancy, pregnant women after ET treatment.

**
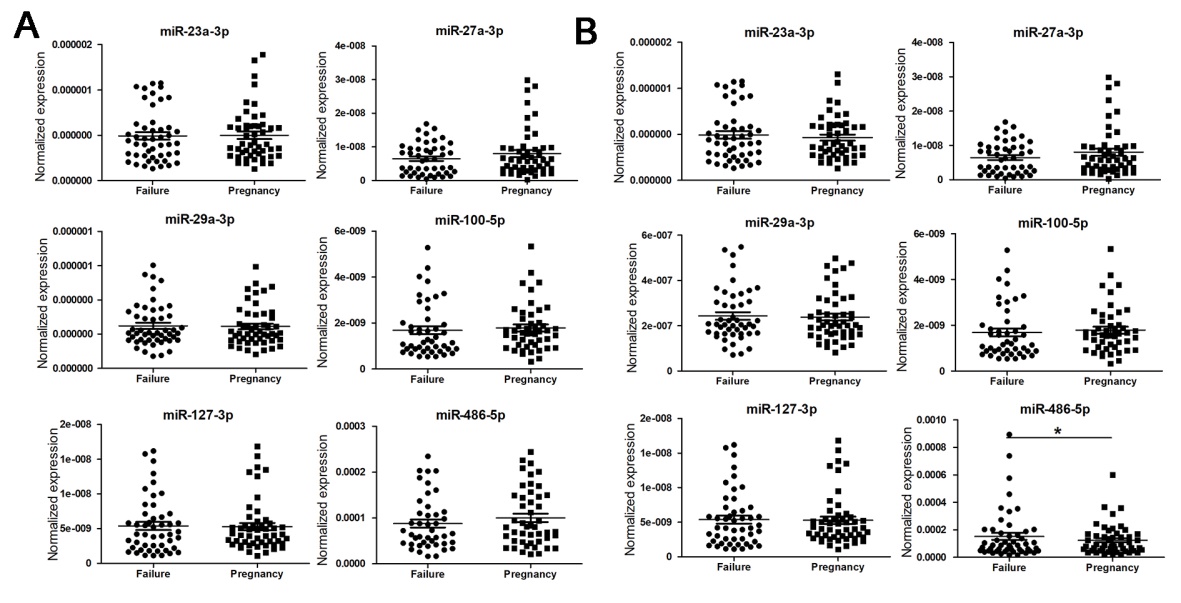
**

**FIGURE S3**

Comparison of relative levels of miR-23a-3p, miR-27a-3p, miR-29a-3p, miR-100-5p, miR-127-3p and miR-486-5p in plasma (A) and serum (B) of peripheral blood between pregnant women (n=22) and non-pregnant women (n=21) after the second, third or fourth-time embryo transfer in a IVF-ET cycle. Failure, non-pregnant women after ET treatment; Pregnancy, pregnant women after ET treatment.

**
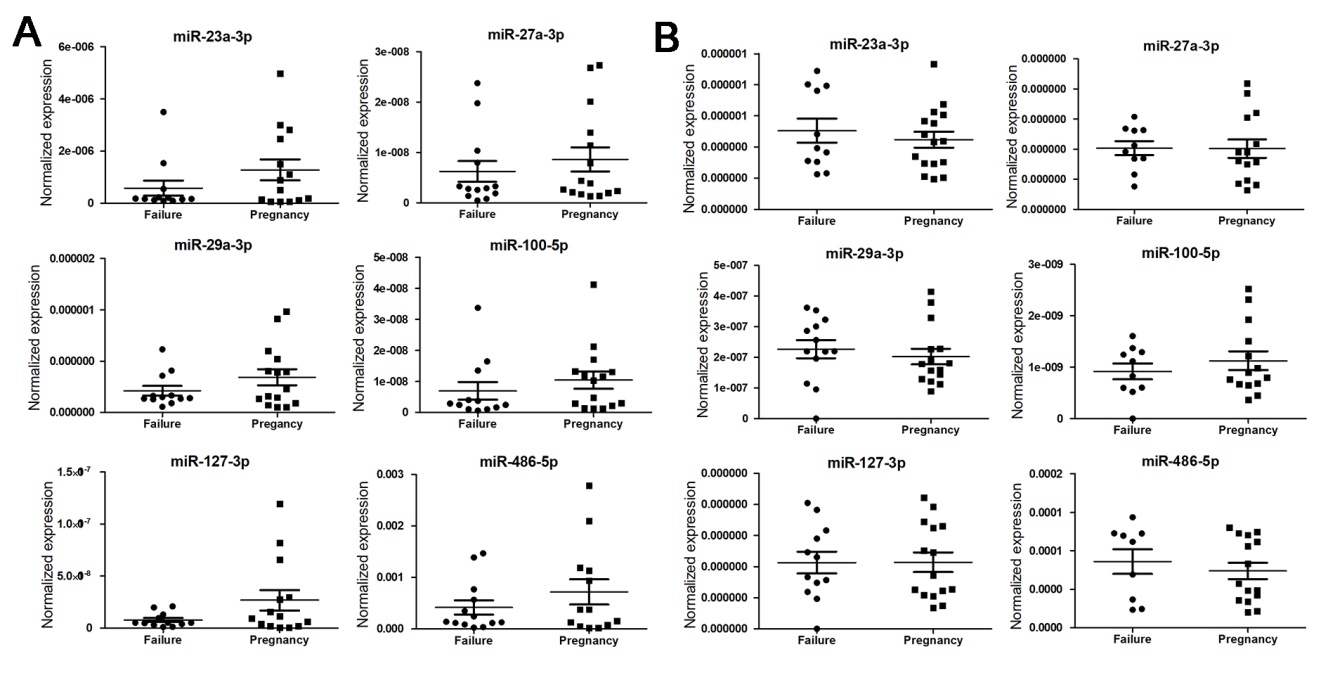
**

**FIGURE S4**

Predictive value assessment by Receiver Operating Characteristic analysis. (A) ROC curve of miRs for the Outcome of IVF-ET. Curves of Model 1 (combined 6 miRs, miR-23a-3p, miR-27a-3p, miR-29a-3p, miR-100-5p, miR-127-3p and miR-486-5p) and Model 2 (combined 2 miRs, miR-127a-3p and miR-486-5p) for peripheral blood plasma; (B) ROC curves of miRs (combined 6 miRs) for peripheral blood serum.

**
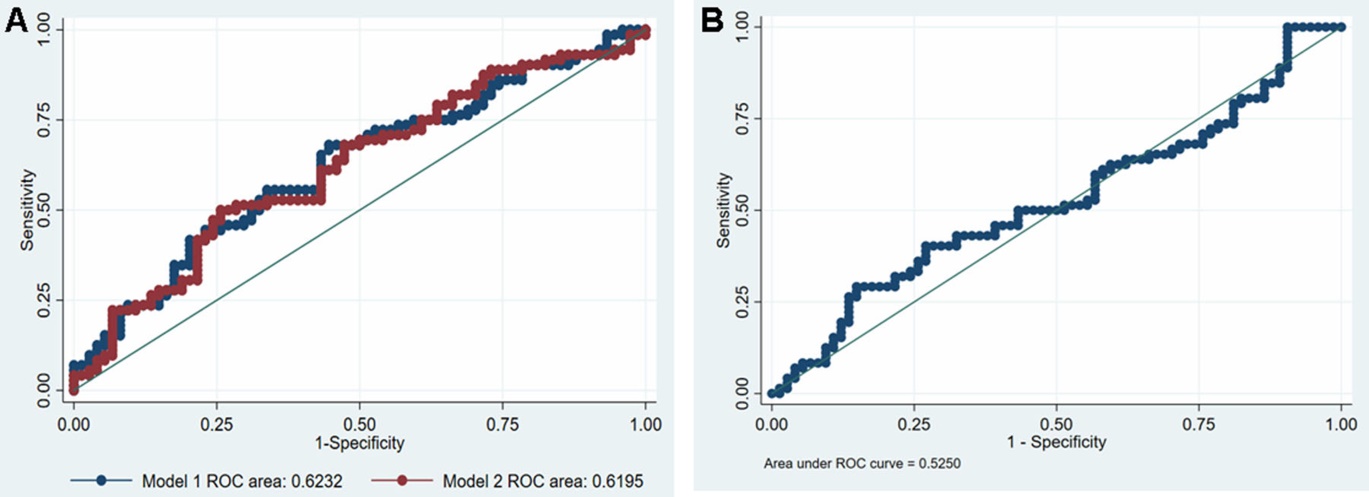
**

**TABLE S1**

**Deep-sequencing results of the differential villus expression of miRNAs in recurrent miscarriage (RM) patients compared to that in normal pregnant (NP) women**

| **miRNA** | **Fold Change (RM vs NP)** | ***p* value** | **Style** |
| --- | --- | --- | --- |
| miR-23a-3p | 1.129035971 | 0.00205531 | Up |
| miR-27a-3p | 1.121637961 | 0.00055181 | Up |
| miR-29a-3p | 1.067498823 | 0.03851844 | Up |
| miR-100-5p | 3.70431902 | 0.00000000 | Up |
| miR-127-3p | 0.88568925 | 0.02446700 | Down |
| miR-486-5p | 0.30897628 | 0.03455981 | Down |

**TABLE S2**

**The sequence of probes used in n Situ Hybridization (ISH) analysis (5′ to 3′):**

| **Probe** | **Sequence** | **Concentration** |
| --- | --- | --- |
| Scramble miR | DIG/ GTGTAACACGTCTATACGCCCA /DIG | 2 μM |
| miR-23a-3p | DIG/ GGAAATCCCTGGCAATGTGAT /DIG | 2 μM |
| miR-27a-3p | DIG/ GCGGAACTTAGCCACTGTGAA /DIG | 2 μM |
| miR-29a-3p | DIG/ TAACCGATTTCAGATGGTGCTA /DIG | 2 μM |
| miR-100-5p | DIG/ CACAAGTTCGGATCTACGGGTT /DIG | 2 μM |
| miR-127-3p | DIG/ AGCCAAGCTCAGACGGATCCGA /DIG | 2 μM |
| miR-486-5p | DIG/ CTCGGGGCAGCTCAGTACAGGA /DIG | 2 μM |

**Table S3**

**Clinical characteristic of 2 recurrent miscarriage (RM) patients and 2 normal pregnant (NP) women whose villus and decidua tissues were used for IHC assay**

| **Group** | **Sample No** | **Age**  **(years)** | **Gestational week** | **Childbearing history** | **Miscarriage**  **history** |
| --- | --- | --- | --- | --- | --- |
| RM | RM1 | 29 | 9 | 0 | 2 |
|  | RM2 | 42 | 11 | 2 | 3 |
| NP | NP1 | 32 | 9 | 1 | 0 |
|  | NP2 | 30 | 8 | 0 | 0 |

**Table S4**

**Association between the peripheral blood levels of miRs and the clinical outcome of IVF-ET by using logistic regression model**

|  | **Model 1^a^** | | | **Model 2 ^b^** | |
| --- | --- | --- | --- | --- | --- |
|  | **OR (95%CI)** | ***P* value** | **OR (95%CI)** | | ***P* value** |
| **In plasma of peripheral blood** | | | | | |
| miR-23a-3p | 1.17 (0.71-1.91) | 0.55 | N/A | | N/A |
| miR-27a-3p | 0.96 (0.72-1.27) | 0.77 | N/A | | N/A |
| miR-29a-3p | 0.74 (0.40-1.36) | 0.33 | N/A | | N/A |
| miR-100-5p | 1.11 (0.87-1.43) | 0.39 | N/A | | N/A |
| miR-127-3p | 0.67 (0.44-1.02) | 0.06 | 0.59(0.38-0.91) | | 0.02 |
| miR-486-5p | 1.40 (1.03-1.92) | 0.03 | 1.58(1.02-2.43) | | 0.04 |
| **In serum of peripheral blood** | | | | | |
| miR-23a-3p | 1.00 (0.51-1.91) | 0.97 | N/A | | N/A |
| miR-27a-3p | 1.08 (0.79-1.47) | 0.63 | N/A | | N/A |
| miR-29a-3p | 0.77 (0.41-1.47) | 0.43 | N/A | | N/A |
| miR-100-5p | 1.06 (0.84-1.34) | 0.64 | N/A | | N/A |
| miR-127-3p | 0.99 (0.70-1.40) | 0.94 | N/A | | N/A |
| miR-486-5p | 1.05 (0.76-1.45) | 0.76 | N/A | | N/A |

^a^ all of 6 miRs were used as potential predictive indexes.

**^b^** only 2 miRs (miR-127-3p and miR-486-5p) were used as potential predictive indexes.

**TABLE S5**

**ROC analysis in prediction for outcome of IVF-ET by the selected miRs in peripheral blood serum**

| **Roc Index** | **Prediction for outcome of IVF-ET**  **by combination of the six miRs** |
| --- | --- |
| AuROC (95% CI) | 0.53（0.44-0.61） |
| Sensitivity (%) | 27.8 |
| Specificity (%) | 85.1 |
| Positive predictive value (%) | 64.5 |
| Negative predictive value (%) | 54.8 |
| Correctly classified (%) | 56.9 |
| Cut-off value | 0.523 |

**TABLE S6**

**ROC analysis in prediction for outcome of IVF-ET by the six miRNAs in plasma when taking the history of IVF-ET treatment into account**

| **Roc index** | **Combination of the six miRNAs** | **Combination of miR-127a-3p and miR-486-5p** | **Models stratification by the times of transplantation** | |
| --- | --- | --- | --- | --- |
|  |  |  | **One time** | **More than one time** |
| AuROC (95% CI)* | 0.62 (0.52, 0.71) | 0.61 (0.52, 0.70) | 0.64 (0.54, 0.75) | 0.68 (0.50, 0.85) |
| Sensitivity (%) | 68.1 | 50.0 | 62.8 | 100 |
| Specificity (%) | 51.4 | 70.0 | 62.8 | 42.1 |
| Positive predictive value (%) | 59.0 | 63.2 | 62.8 | 65.6 |
| Negative predictive value (%) | 61.0 | 57.7 | 62.8 | 100 |
| Correctly classified (%) | 59.9 | 59.9 | 62.8 | 72.5 |
| Cut-off value | 0.48^a^ | 0.54**^b^** | 0.49 | 0.323 |

***** No significant difference in ROC area between Model 1 and Model 2

^a^ Model 1, full model, Cut-off value =0.396，(miR-23a-3p, miR-27a-3p, miR-29a-3p, miR-100-5p, miR-127a-3p, miR-486-5p)（16.30203,19.38028, 16.29351, 17.94127, 19.86086, 9.8526）

**^b^** Model 2, reduced model, Cut-off value=0.382, (miR-127a-3p, miR-486-5p) (19.12003, 8.878942)

**TABLE S7**

**ROC analysis in prediction for outcome of IVF-ET by the six miRNAs in serum when taking the history of IVF-ET treatment into account**

| **Roc index** | **Combination of the six miRNAs** | **Models stratification by the times of transplantation** | |
| --- | --- | --- | --- |
|  |  | **One time** | **More than one time** |
| AuROC (95% CI)* | 0.52 (0.42, 0.61) | 0.52 (0.41, 0.63) | 0.74 (0.58, 0.9) |
| Sensitivity (%) | 12.5 | 23.5 | 57.1 |
| Specificity (%) | 97.1 | 86.3 | 89.5 |
| Positive predictive value (%) | 81.8 | 63.2 | 85.7 |
| Negative predictive value (%) | 51.9 | 53.0 | 65.4 |
| Correctly classified (%) | 54.2 | 54.9 | 72.5 |
| Cut-off value | 0.554^a^ | 0.53 | 0.622 |

***** No significant difference in ROC area between Model 1 and Model 2

^a^ Model 1, Cut-off value =0.213，(miR-23a-3p, miR-27a-3p, miR-29a-3p, miR-100-5p, miR-127a-3p, miR-486-5p)（6.312687, 6.228964, 13.70655, 13.10668, 9.521886, 4.800646）
